# Physical, Chemical and Functional Attributes of *Neera* Honey Infused Extrudates

**DOI:** 10.3390/bioengineering10010114

**Published:** 2023-01-13

**Authors:** Ravi Pandiselvam, Liya T. Joseph, M. R. Manikantan, Anandu Chandra Khanashyam, P. P. Shameena Beegum, S. V. Ramesh, D. Balasubramanian, S. Neenu, Murali Gopal, A. C. Mathew, K. B. Hebbar

**Affiliations:** 1 Physiology, Biochemistry and Post-Harvest Technology Division, ICAR—Central Plantation Crops Research Institute, Kasaragod 671124, Kerala, India; 2 Department of Food Science and Technology, Kerala University of Fisheries and Ocean Studies, Panangad Road, Madavana, Junction, Kochi 682506, Kerala, India; 3 Department of Food Science and Technology, Kasetsart University, 50 Ngamwongwan Road, Ladyao, Chatuchak, Bangkok 10900, Thailand; 4 ICAR—Directorate of Cashew Research, Karnataka 574202, Puttur, India; 5 Crop Production Division, ICAR—Central Plantation Crops Research Institute, Kasaragod 671124, Kerala, India

**Keywords:** extrusion, *neera*, valorization, coconut sugar, functional properties, snacks

## Abstract

Owing to the demand for the consumption of healthy extrudates, this study explored the infusion of *neera* (coconut inflorescence sap) honey in rice flour, corn flour and coconut milk residue blend-based extrudates. *Neera* honey, the concentrated coconut inflorescence sap, has numerous nutrients and a natural source of essential vitamins. Hence, the potential of *neera* honey as a biofortifying compound for the production of healthy extrudates was investigated. The rice and corn based extrudates supplemented with different concentration of *neera* honey have been prepared until the mix reaches 16 and 20% (w.b.) of feed moisture. Effect of addition of *neera* honey on the physical properties (expansion ratio, bulk density, specific length), functional properties (water absorption, water solubility, oil absorption), biochemical properties (total carbohydrates, total sugar, reducing sugar, phenolics, flavonoids, antioxidants), color parameters(L*, a*, b*), proximate compositions (moisture content, ash, protein, fat) and mineral profile of extrudates were recorded. Results suggest that addition of *neera* honey had a significant (*p* ˂ 0.05) impact on all the physico-chemical parameters evaluated. Incorporation of *neera* honey (feed moisture −20%) resulted in extrudates with less expansion, high bulk density and specific length, having high sugar, protein, phenolics, vitamin C and antioxidant activity. The combination of 60% rice flour + 25% corn flour +15% coconut milk residue samples infused with *neera* honey upto 16% feed moisture was found suitable for the preparation of nutritious extrudates based on functional characterization and minerals evaluation.

## 1. Introduction

In the present context, food businesses are being pressed to create more sustainable and eco-friendly production methods. Snack food is an integral component of eating habit of majority of the world’s population [1]. Snack food is generally eaten between meals; however, nowadays the snack food has attained the state of a main meal for most of the people. Snacks come in variety of forms, including packaged snacks and other processed foods. Commercially available extrudates-based snacks are commonly made from rice and corn flour with the addition of table sugar (sucrose). Snacking has a dual impact on human health. It helps in controlling hunger and may help to reduce excess calorie intake, but continuous consumption of the snacks can lead to obesity, diabetes, heart attack and hypertension. Therefore, incorporation of nutrient rich components in the extrudates is imperative to ensure that the snack foods remain healthy.

Various kinds of processing methods are followed for the development of variety of snack foods and one among them is extrusion cooking. Extrusion technology is the most economical and adaptable method for the formulation of novel cereal-based snacks in different textures and shapes [2]. Extrusion cooking has become popular because of its versatility, high productivity, energy efficiency, short time cooking, better product quality and low operating cost [3].Extrudates characterized with a low bulk density and a high expansion index showed higher overall acceptability.

Corn and rice flour are commonly used as raw materials for the production of extrudates. Though corn and rice forms a staple food across the world, both are rich in carbohydrates and are devoid of multiple health promoting functional biomolecules. Hence, the present study aims to incorporate the *neera* honey and coconut milk residue (CMR) as ingredients for the production of nutritious extrudates.

*Neera* or inflorescence sap of coconut is a healthy nutritious drink. Coconut sap honey is a value-added product derived from coconut inflorescence sap with pH 6.0 to 6.5. The preparation of honey is same as that of jaggery however, care must be taken to avoid prolonged heating which leads to charring and change in color [4]. *Neera* honey is rich in Potassium, Magnesium, Zinc, and Iron and is a natural source of 12 essential vitamin B complex and vitamin C. Fructose (38–40%) and glucose (33–35%) are the two primary sugars present in coconut *neera* honey. *Neera* honey has a low glycemic index (GI) and is diabetic friendly. Moreover, *neera* honey has high sugar content and low glass transition temperatures which result in its increased stickiness. Coconut milk residue (CMR) is a by-product of coconut processing industries while producing virgin coconut oil, coconut milk powder, coconut milk yogurt, and flavored coconut milk [5]. The crude fiber and total dietary fiber contents of CMR flour are 25.51% and 46.50%, respectively. CMR has remained an underutilized by product so far and is either used as animal feed or discarded as waste [6]. Incorporation of coconut milk fiber enhances the physiological and nutritional value of food [7]. The food products with high fiber coconut flour have potential to lower the serum cholesterol and increased fecal bulk. CMR has numerous healing benefits, such asanti colon cancer, anti-diabetic and is good for patients suffering from cardiac diseases [8]. Hence, there is a huge potential to utilize the CMR in the development of ready-to-eat food products [5]. CMR can be incorporated with rice and corn mixture to produce healthy snack food by resorting to the process of extrusion.

These by-products can be used as feedstock for extrusion technology, which can turn them into a variety of manufactured foods that are ready for consumption, such as morning cereals or snack food. Research on incorporation of these by-products into other convenience food products such as extruded produce can be helpful in developing practical measures to enhance the nutritional attributes and consumer acceptance. Hence, this study was conducted to develop rice and corn based extruded product with the infusion of CMR and *neera* honey and to evaluate physical, functional, biochemical, color, proximate composition and mineral characteristics of extrudates developed. Infusion of *neera* honey could avoid seasoning of extrudates due to its natural sweet taste. To the best of our knowledge no study was conducted using *neera* honey for the production of extrudates. Hence, incorporation of *neera* honey in extruded snacks was expected to offer a novel perspective and an alternative nutritious snack production.

## 2. Materials and Methods

### 2.1. Raw Materials

The raw materials, such as corn flour (CF) and rice flour (RF) were purchased from the local market in Kasaragod, India. The CMR used in this study was collected from the Agro Processing Complex of ICAR-CPCRI, Kerala, India [9]. In brief, coconut milk was separated from freshly harvested mature coconuts (12 months old) using a double screw milk expeller. Following three consecutive extractions, the residue obtained was dried at 60–65 °C in a tray dryer to moisture content of 2.5–4% (w.b.) and then sieved through astandard mesh (0.251 mm) using a sieve shaker. For the preparation of *neera* honey (NH), freshly tapped *neera* (pH 7 to 7.5) collected from coconut plantation in ICAR—CPCRI, using coco-sap chiller, was immediately processed at Agro Processing Complex. The collected *neera* was strained to eliminate undesirable contaminantsand then concentrated in a virgin coconut oil cooker at 115 °C until the total soluble solids (TSS) reached between 60 and 70° brix [10]. This concentrate was then collected, placed into a glass beaker, and kept in the refrigerator until further use.

### 2.2. Preparation of Raw Materials for Extrusion

Nine feed flour combinations comprising rice flour (RF), corn flour (CF), coconut milk residue (CMR) and *neera* honey (NH) were prepared (Table 1). The combinations were mixed and sieved through British Standard (BS) Sieve of 14 mesh size (1 mm). Nine different combinations were (1) 100% RF + 16% feed moisture with water, (2) 100% RF + 16% feed moisture with NH, (3) 100% CF + 16% feed moisture with water, (4) 100% CF +16% feed moisture with NH, (5) 50% RF + 50% CF + 16% feed moisture with NH, (6) 50% RF + 50% CF + 16% feed moisture with water, (7) 60% RF + 25% CF + 15% CMR + 16% feed moisture with NH, (8) 100% RF + 20% feed moisture with NH, and (9) 85% RF + 15% CMR + 20% feed moisture with NH prepared. The feed flours were added with *neera* honey until it reaches the desirable moisture content (16% and 20%). The feed moisture content was selected based on previous reports [4,11]. In the case of incorporation of CMR, 15% was only added in accordance with our previous study [4].

### 2.3. Extrusion

The extrusion was carried out in a co-rotating twin-screw extruder (KK Life Sciences Chennai, Tamil Nadu, India) with a 7.5 HP motor. The set temperature, screw speed and feed rate of the extruder were 110 °C, 220 rpm and 15 kg/h, respectively. The extruder die diameter was 4 mm, and L/D ratio of the screw was 24.5. During extrusion, the first, second, third, and fourth zones’ temperatures were kept at 35 °C, 50 °C, 90 °C, and 110 °C, respectively. The resulting extrudates were dried for 20 min at 60 °C in a tray dryer.

### 2.4. Measurement of Physical Properties

The analysis of physical properties of extrudates included expansion ratio, bulk density, and specific length by following the standard procedures [4,11].

### 2.5. Measurement of Functional Properties

Functional properties of the extrudates measured in this study include water absorption, water solubility and oil absorption.

The water absorption index (WAI) was determined Anderson et al. [12] used for corn grits, with slight adjustments. For the study, 0.5 g of the powdered samples was combined with 15 mL of distilled water and mixed utilizing a vortex mixer for 2 min (Riviera Glass Pvt., Ltd., Mumbai, India). After letting the mixture stand for 30 min, it was centrifuged at 6000 rpm for 30 min (M/s HermleLabortechnik GmbH, Wehingen, Germany). The supernatant liquid was collected carefully into a tapered evaporating dish, and the WAI was determined from the weight of the residual gel.

The water solubility index (WSI) is the amount of polysaccharides that is released from a granule during hydration and is represented by the weight of dry solids in the supernatant. The additional moisture present in the supernatant liquid was removed by evaporation with the help of a tapered evaporating dish. The WSI, which is expressed as a percentage, was calculated by dividing the weight of the dry solids recovered by evaporating the WAI test supernatant to the weight of the starting sample (0.5 g) [4].

The measurement of oil absorption index (OAI) was performed similar to WAI with a little modification. Approximately 0.5 g of powdered samples was mixed in a 5 mL of oil in a test tube and the mixtures vortexed for 2 min and kept for 10 min. It was followed by centrifugation and recovery of sediment and weighing. The analysis was conducted in triplicates.

### 2.6. Measurement of Color Properties

The psychometric index of lightness (L*) and two color coordinates, a* and b* of the extrudate samples were assessed using CIELAB scale at 10° observer and D65 illuminant (Konica Minolta CM-2500c, Tokyo, Japan).

### 2.7. Measurement of Biochemical Properties

Extrudates and raw materials were extracted with 80% ethanol, and the total carbohydrate, total sugars, and reducing sugar were estimated following the standard biochemical techniques. The phenol sulphuric acid method [13] was used to estimate total soluble sugar and total carbohydrate, whereas, Nelson Somogyi’s method was used to measure reducing sugar contents [14].

Proximate analysis, of raw materials and *neera* honey based extrudates, including moisture (AOAC 934.01), ash (AOAC 938.08), protein (AOAC 2001.11), and fat (AOAC 920.58), was determined. The total phenolic content was determined using the Follin–Ciocalteu (FC) test, as reported by Seneviratne et al. [15], and was expressed as mg gallic acid equivalent (GAE) per 100 g extrudates. The total flavonoid content (TFC) was calculated using the method described by Zhishen et al. [16].The volumetric approach used by Harris and Ray [17] was used to quantify the amount of vitamin C (ascorbic acid). While the antioxidant properties of the samples were measured by two techniques: FRAP (Ferric reducing antioxidant power) and CUPRAC (Cupric ion reducing antioxidant capacity). CUPRAC of the extrudate was determined by following the procedure of Apak et al. [18], and FRAP was performed according to Benzie and Strain [19]. Results from both the techniques were represented in mg TE (Trolox equivalent) per 100 g of extrudates, where Trolox served as the standard.

### 2.8. Estimation of Mineral Contents

Total Phosphorus (P) concentration of the sample was measured on a UV–visible spectrophotometer (UV-1601, Shimadzu, Tokyo, Japan) after developing yellow colour by vanadate molybdate method [20,21]. The potassium (K) and sodium (Na) concentrations were measured in a flame photometer (model CL-378, Elico Ltd., Telangana, India). The total Fe, Zn, Cu and Mn were estimated by Atomic Absorption Spectrophotometer (Thermo Scientific iCE 3000 series, Boston, MA, USA) using AOAC procedures.

### 2.9. Statistical Analysis

All the experiments including determination of various parameters were conducted in triplicates unless mentioned otherwise. All data were expressed as means ± Standard deviation (SD). The influence of adding *neera* honey and CMR to rice and corn based extrudates on the properties of extrudates was analyzed using completely randomized design with the ANNOVA procedure in SAS Ver.9.3 (SAS Institute Inc., 2011, Cary, NC, USA).

## 3. Results

### 3.1. Raw Material Analysis

#### 3.1.1. Functional Properties and Proximate Composition of Raw Materials Used for Preparation of Extrudates

Water absorption index (WAI) of the rice flour (RF), corn flour (CF) and coconut milk residue (CMR) were 2.39 ± 0.05, 2.18 ± 0.11 and 7.12 ± 0.39, respectively (Table 2). *Neera* honey (NH) was fully soluble in water. A flour’s capacity to bond with water molecules is represented by the WAI [22,23]. It is a result of high number of hydrophilic groups present in the starch granules and can affect the softness and viscosity of the food products. WAI can be affected by the negative charges on the phosphate groups in amylopectin which can cause an increase in water binding ability [24]. The presence of carbohydrates and proteins, frequently promote significant hydrogen bonding due to their polar or charged side chains [25]. The values determined by Preethi et al., [11] for WAI of RF (2.70 ± 0.04) and CF (1.81 ± 0.02) were similar to the resulted presented herein. WAI of CMR (3.58–8.03 mL/g) as reported in our previous study [5] was also in the range of the observed results. The WAI of CMR were reported to be significantly higher than RF and CF, this may be due to the large particle size of CMR as higher particle size tends to lower the value of WAI [26,27].

Water solubility index (WSI) showed higher value in NH (89.22 ± 0.73%) when compared to RF (1.19 ± 0.56%), CF (5.87 ± 2.10%), and CMR (12.03 ± 0.12%). However, the results of the present study were accordance with previous reports [4,11]. The starch granules present in the raw materials RF, CF and CMR are hygroscopic in nature rendering them water soluble and it is represented by the WSI value [11]. A low WSI mean lower ability to preserve food structure, whereas a higher WSI suggests a higher value of stickiness and adhesiveness in food products [24]. NH is hydrophilic in nature and it showed 90% solubility in water. Oil absorption index (OAI) was found to be high in CMR (1.31 ± 0.03) when compared to RF (1.20 ± 0.05), CF (1.21 ± 0.01) and NH (1.30 ± 0.05).The relatively less OAI could be due the presence of starch in RF, CF and CMR, which has the capacity to hold and absorb oil by its non-polar side chains of the chemical components [28]. Similar results of OAI for RF (2.08 ± 0.02) and CF (2.31 ± 0.27) were reported by Preethi et al. (2021) [11].

The proximate composition analysis was carried out to evaluate the moisture, ash, fat, and protein content of the raw materials. The analysis indicated that moisture content (MC) of the NH was found to be high (29.96 ± 0.00%) followed by RF (9.30 ± 0.09%), CF (8.38 ± 0.48%) and CMR (5.72 ± 0.04%), because NH contains almost 30% water (Table 2). It was observed that water molecule tend to remain bound with cereal grains and it does notevaporate as fast as observed in NH during heating. The fat and ash content of the *neera* honey was significantly less than that of RF, CF and CMR because NH predominantly contains sucrose. The protein content of NH was too low (1.41 ± 0.21%) compared to RF (9.44 ± 1.01%), but CF (7.291 ± 0.49%) and CMR (7.297 ± 0.21%) showed an almost comparable amount of protein. Asaduzzaman et al. [29], reported natural honey contains 15.42 ± 0.37 g/100 g MC, 0.36 ± 0.08 g/100 g ash content, 0.20 ± 0.10 g/100 g fat and 0.68 ± 0.29 g/100 g protein.

#### 3.1.2. Biochemical Composition of Raw Materials Used for Preparation of Extrudates

The biochemical composition *viz*, total carbohydrate (CHO), total sugar, reducing sugar, phenols, flavonoids and antioxidant activity (CUPRAC, FRAP) were analyzed for the raw material. Biochemical analysis reveals that the highest total carbohydrate percentage was present in RF (92.03^A^ ± 3.11) followed by CF, CMR and NH (49.29^B^ ± 2.23, 32.09^C^ ± 4.11, 30.62^C^ ± 7.26, respectively) (Table 3). The low total carbohydrate content reported in NH is due to the fact that starch is the major storage carbohydrate in cereals and second largest most abundant polysaccharide. However, NH was reported to contain significantly higher amount of total sugar and reducing sugar than other raw materials used in this study. Asaduzzaman et al. [29] recorded similar results for honey samples. Nevertheless, NH is rich in ascorbic acid, total phenol content, flavonoids and antioxidant activity.

#### 3.1.3. Mineral Profile of Raw Materials Used for Preparation of Extrudates

The analysis of minerals revealed that RF contains high amount of magnesium and low in calcium, potassium, sodium and zinc. Corn flour contains high amount of phosphorus, sulphur iron and zinc and low in magnesium and copper (Table 4). CMR contains high amount of calcium, magnesium, and copper. CMR contains almost every mineral in appreciable quantity. *Neera* honey was rich in potassium and sodium and low in phosphorus, iron and manganese (Table 4).

Similar results were reported by Preethi et al., [11] in cashew apple pomace powder that contains major minerals such as calcium, phosphorus, magnesium, and potassium in RF (12.70 ± 2.47 mg/g, 8.00 ± 1.53 mg/g, 0.18 ± 0.11 mg/g, 0.30 ± 0.02 mg/g) and CF (22.50 ± 1.37 mg/g, 12.33 ± 1.53 mg/g, 1.76 ± 0.06 mg/g, 2.90 ± 0.06 mg/g). Asaduzzaman et al. [29] recorded that the honey samples have the following composition: calcium (3.0260 ppm), phosphorus (2.0300 ppm), magnesium (0.7069 ppm), potassium (0.0569 ppm) and sodium (1.6020 ppm),and (trace minerals) in RF were iron (9.50 ± 0.72 mg/g), manganese (0.61 ± 0.07 mg/g), zinc (1.13 ± 0.09 mg/g), and copper (16.95 ± 0.37 mg/g), whereas corresponding values for CF were 9.98 ± 0.27 mg/g, 1.65 ± 0.05 mg/g, 1.73 ± 2.48 mg/g, 20.75 ± 0.50 mg/g. Similarly, the trace elements of honey were recorded as 0.2050 ppm, 0.0059 ppm, 0.0208 ppm, 0.0427 ppm [29].

### 3.2. Neera Honey Incorporated Extrudates

**Figure 1:**
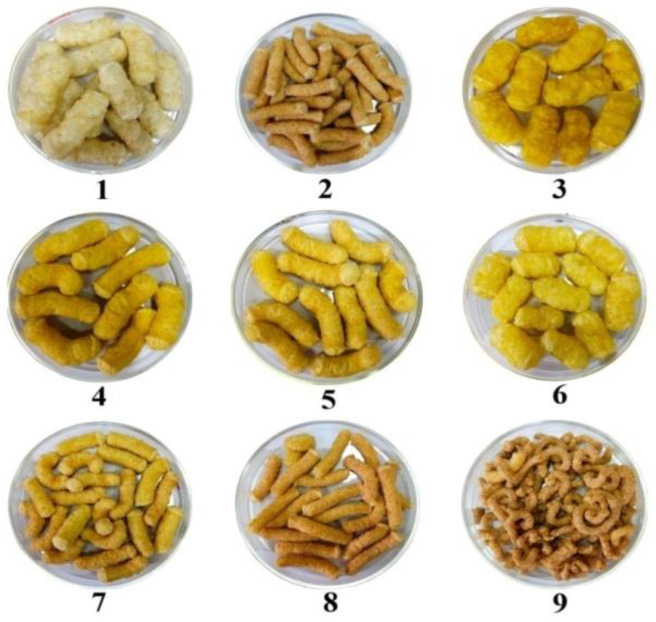
Different types of extrudates prepared with rice flour, corn flour, coconut milk residue and *neera* honey (**1**) 100% RF + 16% FM (water), (**2**) 100% RF + 16% FM (NH), (**3**) 100% CF + 16% FM (water), (**4**) 100% CF +16% FM (NH), (**5**) 50% RF + 50% CF + 16% FM (NH), (**6**) 50% RF + 50% CF + 16% FM (water), (**7**) 60% RF + 25% CF + 15% CMR + 16% FM (NH), (**8**) 100% RF + 20% FM (NH), (**9**) 85% RF + 15% CMR + 20% FM (NH).

Rice and corn based extrudates were prepared by incorporating CMR and *neera* honey. Rice flour and corn flour in different proportions was mixed with 15% CMR, 16 and 20% feed moisture with *neera* honey and 16% feed moisture with water (Figure 1). During extrusion the flour is initially converted in to a viscoelastic bulk due the high pressure, shear forces, and temperature [30]. This step is followed by the entrapment of small air bubbles in the mass called nucleation where the entrapped air bubbles later expand in size as a result of the fast moisture evaporation termed as bubble growth.Finally, as the viscoelastic mass cools, the expansion of these bubbles stops. The degree of expansion depends on structural damage of the granules of gelatinized starch during extrusion [30]. The different morphology of the resulting extrudates can be related to the variations in the expansion ratio. The expansion ratio is the ratio of the extrudate’s cross-sectional diameter to that of the die which serves as a measure of how much the extrudate has expanded [5]. The product expansion, or extrudate expansion, is a crucial characteristic describing the product quality in extrusion process and is directly proportional to the degree of cook. According to Yadav et al. [31], the extrudates’ expansion ratio varies according to the inherent physicochemical characteristics of the flour and the extrusion conditions such as temperature, moisture content and screw speed. Studies have also shown that the presence of high level of feed moisture, fiber or protein could negatively affect the expansion ratio [32], which may explain the differences in morphology of the extrudates.

#### 3.2.1. Physical Properties of *Neera* Honey Infused Extrudates

Physical properties *viz*, specific length, expansion ratio and bulk density of *neera* honey incorporated rice flour, corn flour and coconut milk residue based extrudates are depicted in Table 5.

##### Expansion Ratio

**Figure 2:**
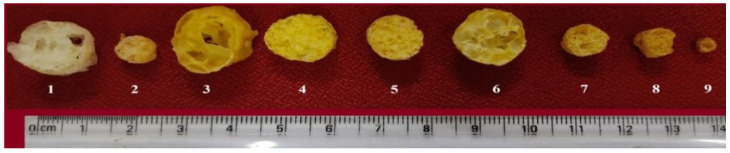
Cross section view of extrudates prepared with rice flour, corn flour, coconut milk residue and *neera* honey (**1**) 100% RF + 16% FM (water), (**2**) 100% RF + 16% FM (NH), (**3**) 100% CF + 16% FM (water), (**4**) 100% CF +16% FM (NH), (**5**) 50% RF + 50% CF + 16% FM (NH), (**6**) 50% RF + 50% CF + 16% FM (water), (**7**) 60% RF + 25% CF + 15% CMR + 16% FM (NH), (**8**) 100% RF + 20% FM (NH), (**9**) 85% RF + 15% CMR + 20% FM (NH).

Expansion ratio (ER) is an important characteristic feature of extrudates. It measures the degree of puffing of extrudates [33]. The ER of the extrudates ranged from 1.64 ± 0.09 to 4.02 ± 0.19 (Table 5). The ER was found to be high (4.02 ± 0.19) in an extrudate made using F_3_ followed by the combination F_6_ (3.78 ± 0.02). The amount of starch, as well as the size, quantity, and distribution of air cells within the extrusion, affect ER. The increased ER could be attributed to the porous and sponge-like structure formed by steam bubbles as a result of quick release of pressure after exiting the die [34]. The lowest ER was obtained from F9 (Figure 2). The addition of *neera* honey had a substantial impact on the ER. Increasing level of NH resulted in decreased ER due to the sugars in NH [35]. When compared to the other combinations, the extrudates that incorporated 20% *neera* honey exhibited an extremely low ER. *Neera* honey inclusion raises sugar levels, which compete with starch granules thereby affecting the ER. ER of honey incorporated snack was found to be 2.01 [36]. The extrudates brand available in the market has the ER of 3.2. It was observed that incorporation of *neera* honey in RF, CF, and CMR blends significantly (*p* < 0.001) affected the ER.

##### Bulk Density

The bulk density of the extrudates infused with *neera* honey showed an increase compared to the others. Bulk density values range from 0.079 ± 0.001 g/mL to 0.281 ± 0.007 g/mL. Bulk density is inversely proportional to expansion ratio, because when expansion ratio increases bulk density decreases and vice versa. The bulk density of extrudates increased with the increased concentration of honey. The expansion ratio of the extrudates prepared using the combination of F_3_ was observed to be high (4.02 ± 0.19) thereby a lowin its bulk density (0.079 ± 0.001 g/mL). Sugar contents tend to decrease water activity, which impacted bulk density of extrudates [37]. According to Case, et al. [38], a rise in die temperature led to an increase in the degree of superheating of water in the extruder, which encouraged bubble formation and a drop in melt viscosity. BD of the honey incorporated snack is 187 kg/m^3^ [36]. Thus, incorporation of *neera* honey in RF, CF, and CMR blend significantly (*p* < 0.001) affected the BD.

##### Specific Length

The specific length (SL) of extrudates correlates with length and weight measured in terms of axial expansion [33]. It was observed that the incorporation of NH in RF and CF increased the SL of extrudates. The range of SL significantly increased from 33.99 ± 1.37 mm/g to 95.51 ± 15.96 mm/g (Table 5). The highest value was recorded (95.51 ± 15.96 mm/g) in the combination of F_9_. The high gelatinization of starch could be attributed for the increase in SL [39]. The lowest SL observed in the combination of F_3_ probably due to the high quantum of starch present in the CF; the puffing of extrudate will be high so that there would be a decrease in the weight of the extrudate. Incorporation of *neera* honey in RF, CF and CMR blend significantly (*p* < 0.001) affected the SL.

#### 3.2.2. Functional Properties of *Neera* Honey Incorporated Extrudates

Functional properties of extrudates, such as water absorption, water solubility and oil absorption determine their suitability for different applications. The functional characteristics of the extrudates are interconnected with the molecular alterations that take place during the extrusion cooking process. Results of functional properties of the extrudates obtained from various combinations are given in Table 6.

##### Water Absorption Index

Water absorption index (WAI) measures the amount of water absorbed by the starch and other compounds in extrudates. It is also referred to as the index of gelatinization. Gelatinized starch has the ability to absorb more quantum of water than the other constituent in the extrudates [33]. WAI of the extrudates ranged from 2.61 ± 0.07 to 7.47 ± 0.25. High WAI was recorded in the extrudate made of F_3_. It may be due to the high starch content present in CF. The lowest value was recorded in the combination of F_9_.Incorporation of CMR and NH which contains only 30% of starch caused the reduction in WAI because CMR contains high amount of fiber as well as NH contains high amount of sugar. On the contrary RF contains almost 90% of starch. An experiment involving barley flour with honey, recorded WAI of 3 to 4.48 [35]. Thus, incorporation of *neera* honey in RF, CF, and CMR blend significantly (*p* < 0.001) affected the WAI.

##### Water Solubility Index

Water solubility index (WSI) is used as an indicator to measure the amount of soluble polysaccharides released from starch and degradation of molecular compounds during extrusion [40]. The WSI calculates the quantity of soluble substances liberated from the starch following extrusion. High WSI reflects the degree of dextrinization and gelatinization, which is a sign of good starch digestibility. WSI of the extrudates ranged from 7.27 ± 3.55% to 24.23 ± 2.28%. The highest WSI was observed in the formulation of F_9_ since 90% of NH is soluble in water. With the incorporation of 20% of NH there was an increase in solubility, also CMR would not tightly bind with water. Starchy component in the combination is provided only by rice flour, during the extrusion process degradation of macromolecules happens and the molecular weight of starch granules decreases causing an increment in WSI [41]. The lowest value of WSI observed in F_6_ could be due to the high amylose content in RF and CF. The amylose–amylose and amylose–amylopectin interactions after starch gelatinization form crystalline structure, thereby reducing the solubility [42]. The WSI ranged from 6.6% to 18.2% in a previous study involving barley flour and honey [35]. Incorporation of *neera* honey in RF, CF and CMR blend significantly (*p* < 0.001) affected the WSI.

##### Oil Absorption Index

Oil absorption index (OAI) measures the oil holding capacity of extrudates. In this study, OAI of nine combinations exhibited only slight differences. The oil absorption values range from 1.41 ± 0.12 mL/g to 1.55 ± 0.06 mL/g. The absorption of oil by the non-polar side chains of the chemical constituents could be responsible for the oil being absorbed by the extrudates. It might be the reason for highest OAI recorded in F_1_. The lowest OAI was recorded in F_7_. The OAI values were found to be lower in samples which contain hydrophilic compounds which retard the absorption of the oil [33].Incorporation of *neera* honey in RF, CF and CMR blend did not significantly affect the OAI.

#### 3.2.3. Color Attributes of *Neera* Honey Incorporated Extrudates

Color is one of the most important and conspicuous characteristics of any food product. The color values of extrudates prepared with and without the addition of *neera* honey are presented in Table 7.

The color parameter L* (luminosity) varied from 37.68 ± 2.31 to 62.46 ± 0.72 on a 1 to 100 scale. The highest L value of extrudates was recorded in the combination F_3_ and lowest was recorded in F_9_. The value was found to increase due to the thermal degradation of NH and it could be reduced with the reduction of NH. Increased L value may be due to the presence of phenolics and other contents in NH. Incorporation of *neera* honey in RF, CF and CMR blend significantly (*p* < 0.001) affected the L* value. The color value a* indicates greenness to redness. The values ranged from −0.09 ± 0.46 to 5.58 ± 0.92. The highest value that was found to be in F_8_ could be due to the low feed moisture, and the lowest value was found in extrudates made of F_3_ owing to bright yellow color it had and the high starch content in CF. Incorporation of *neera* honey in RF, CF and CMR blend significantly (*p* < 0.001) affected the a* value. The color value b* indicates blueness to yellowness. The values (b*) ranged from 9.99 ± 1.73 to 26.73 ± 0.36. Addition of NH leads to increase a* and b* values and samples were darkened due to the high quantity of free monosaccharide present in the honey. The factors that affect the color of the extrudates are browning reaction including Maillard reaction and caramelization which happened when the extrudates have undergone extrusion [43]. Incorporation of *neera* honey in RF, CF, and CMR blend significantly (*p* < 0.001) affected the b* value. A prior study by Bobade, Singh, Sharma, Gupta and Singh [36] in whole wheat flour based extrudate recorded L*, a* and b* values as follows 63.15, 3.25, and 19.35, which is in the range of the extrudates presented herein.

#### 3.2.4. Biochemical Properties of *Neera* Honey Extrudates

The biochemical features of *neera* honey-based extrudates are given in Table 8. Available carbohydrate (CHO) content in the extrudates ranged from 56.49 ± 1.35% to 80.54 ± 5.15%. It was recorded high in F_3_ and lowest in F_1_. The result suggest that in F_2_ there was an increase in carbohydrate content when compared to F_1_ and similarly, CHO content increased in F_4_ when compared to F_3_. However, in F_6_ (63.42 ± 7.61%) and F_5_ (65.32 ± 8.15%), there was no significant change in carbohydrate. Arivalagan et al., (23) report that the available carbohydrate in coconut haustorium extrudates was 71.2 g to 80.1 g. Incorporation of *neera* honey in RF, CF and CMR blend did not significantly affect the total CHO.

Total sugar content of the extrudates prepared with *neera* honey was found significantly high compared to other extrudates. The value ranged from 1.78 ± 0.55% to 36.82 ± 5.72%. The incorporation of 16% and 20% of *neera* honey increased the sugar content from 12.60% to 36.82%. NH based extrudates have a high concentration of soluble sugars, providing natural sweetness to the extrudates that rice and corn-based extrudates are lacking. This suggests that no additional sweeteners are needed for the preparation of extrudates [33]. In coconut haustorium based extrudates, total soluble sugar content was found significantly high compared to control extrudates that were based on rice and maize 5.56% to 13.28% [23]. Incorporation of *neera* honey in RF, CF, and CMR blend significantly (*p* < 0.001) affected the total sugar content. Reducing sugar content of the extrudates ranged from 0.03 ± 0.01% to 2.81 ± 1.31%. As the incorporation of NH increased the quantum of reducing sugar also increased. In F_6_ reducing sugar was recorded as 0.03 ± 0.01% whereas in F_8_ reducing sugar content was high (2.81 ± 1.31%). Incorporation of *neera* honey in RF, CF and CMR blend significantly (*p*-Value 0.0027) affected the reducing sugar content.

#### 3.2.5. Proximate Composition of *Neera* Honey Incorporated Extrudates

Proximate composition of *neera* honey extrudates is presented in Table 9. Moisture content (MC) of the extrudates ranged from 3.76 ± 0.015% to 10.61 ± 3.17%. The highest MC was recorded in the combination F_6_ and lowest value was in F_7_. Product moisture is reported to be inversely correlated with extrusion temperature and directly correlated with feed moisture [32]. The water present in the extrudates were tightly bound and it is difficult to evaporate quickly however water in extrudates incorporated with NH evaporates fast hence it resulted in caramelization causing reduced MC. In the haustorium incorporated extrudates moisture content was significantly reduced from 6.64% to 3.56% [33].

Ash content of the extrudates varied from 0.29 ± 0.13% to 0.89 ± 0.13% representing the mineral composition. Incorporation of NH showed slight increase in ash content. In the coconut haustorium incorporated extrudates ash content significantly increased from 1.26% to 1.90% [33]. Incorporation of *neera* honey in RF, CF, and CMR blend did not significantly affected the ash content. Total fat content ranged from 1.14 ± 0.35% to 4.96 ± 2.72%. Incorporation of NH to 100% RF and 100% CF, a decrease in fat content was recorded. A small amount of lipid could be lost as free oil at the die, however this only happens with high-fat materials, such as whole soy. The development of complexes with amylose or protein is another explanation for the reduced amount of lipids during NF addition. In the coconut haustorium incorporated extrudates fat content ranged from 0.19% to 4.54% [33]. Incorporation of *neera* honey in RF, CF, and CMR blend did not significantly affect the total fat content.

Protein content in the extrudates varied between 7.51 ± 0.14% in F_9_ to 9.23 ± 1.64% in F_7_ and no significant difference was reported in the protein content in any of the extrudates combination. The combination which showed highest protein content could be due to the incorporation of RF, CF, CMR and NH. Even though NH contains only less amount of protein, other raw materials could contribute their characteristics to the extrudates. In the haustorium incorporated extrudates protein content varied between 5.62% to 8.17% [33].

#### 3.2.6. Phenolics, Flavonoids, Vitamin C and Antioxidant Properties of *Neera* Honey Based Extrudates

Phenolic compounds are the major biomolecules that contribute to the antioxidant activity of cereals [44,45]. Total phenolic content (TPC) of the extrudates ranged from 0.161 ± 0.03 mg GAE/100 g to 0.359 ± 0.10 mg GAE/100 g. As the incorporation of NH increased the TPC was also increased. Highest TPC was recorded in a combination F_7_, due to the presence of the raw materials including NH which had highest TPC. The lowest value was recorded in the combination F_3_ which could be due to high temperature or molecular structure alteration leading to the decreased reactivity and extractability (Kumar et al., 2013). In the coconut haustorium study, phenolics content varied between 20.9 to 212.8 mg GAE [33]. Incorporation of *neera* honey in RF, CF and CMR blend significantly affected the phenolic content.

Flavonoids are crucial for maintaining human health because it has potent pharmacological actions and act as radical scavengers [46]. The total flavonoid content (TFC) values of the extrudates varied between 0.177 ± 0.02 mg QE/100 g in F_2_ and 0.528 ± 0.00 mg QE/100 g in F_3_. Incorporation of NH reduced the flavonoid content in the extrudates. The reduction in TFC might be due to molecular structure alteration or high temperature caused during extrusion which led to the decreased reactivity and extractability [35]. In the coconut haustorium study flavonoids content varied between 7.94 to 95.2 mg GAE [33]. Incorporation of *neera* honey in RF, CF and CMR blend significantly affected the flavonoid contents.

The ascorbic acid (Vitamin C) content in the extrudates increased witha proportionate increase in the NH. Ascorbic acid is sensitive to heat and oxidation. Singh, et al. [47] reported that the vitamin retention in extrusion cooking diminishes with rising temperature, faster screw rotation, and increased specific energy input. Additionally, it declines when moisture, feed rate, and die diameter decrease. The ascorbic acid values ranged between 2.34 ± 0.01 mg/100 g in F_3_ and 17.59 ± 0.07 mg/100 g in F_9_. NH is a rich source of vitamin C. Vitamin C is affected during extrusion process due to the temperature, but the effect is not severe as that of the other vitamins. Incorporation of *neera* honey in RF, CF and CMR blend significantly (*p* < 0.001) affected the vitamin C. A similar result in the pattern of vitamin C was also reported by Preethi [11] after extrusion of cashew apple enriched flour.

The antioxidant activities of the extrudates as measured in CUPRAC and FRAP assays were found to be indirect (Table 10). The antioxidant value as measured in CUPRAC assay ranged from 0.80 ± 0.13 mg TE/100 g in F_2_ to 2.97 ± 0.27 mg TE/100 g in F_3_. The extrusion process caused a negative impact on CUPRAC value. Incorporation of *neera* honey in RF, CF and CMR blend significantly (*p* < 0.0005) affected the CUPRAC values. Antioxidant potential of extrudates as measured in FRAP assay increased with an increase in NH incorporation. It varied between 0.048 ± 0.007 mg TE/100 g in F_1_ to 0.076 ± 0.00 mg TE/100 g in F_4_ and F_9_. Incorporation of *neera* honey in RF, CF and CMR blend significantly affected the FRAP assay.

#### 3.2.7. Mineral Composition of *Neera* Honey Incorporated Extrudates

The mineral analysis reveals that the extrudates comprise phosphorus, potassium, sodium, calcium, magnesium, sulphur, iron, manganese, zinc, and copper. It can be divided into two categories major minerals and trace minerals.

Minerals are considered stable during heat treatment. However smaller molecules may be affected by the extrusion process due to the structural changes in larger molecules, which in turn can affect other compounds present in the food. Studies have reported mineral stability during extrusion cooking of cereal grains. Extrusion cooking can improve the absorption of minerals by reducing other factors that inhibit their absorption [48,49]. The Ca content of the extrudates ranged between and 97.74 ± 22.15 mg/100 g in F_1_ and 184.80 ± 22.02 mg/100 g in F_3_. Incorporation of *neera* honey in RF, CF and CMR blend significantly (*p* < 0.001) affected the calcium content.

The P content of the extrudates ranged between 69.47 ± 11.34 mg/100 g in F_2_ and 103.16 ± 2.24 mg/100 g in F_8_ (Table 11). It was found that when the addition of NH increased the P content increased. Incorporation of *neera* honey in RF, CF and CMR blend significantly affected the P content. The value of Mg content of the extrudates ranged between 52.00 ± 0.73 mg/100 g in F_7_ and 97.65 ± 12.76 mg/100 g in F_1_. It was observed that incorporation of NH reduced the quantum of Mg. The K content of the extrudates ranged between 46.84 ± 3.83 mg/100 g in F_1_ and 126.93 ± 10.56 mg/100 g in F_9_. The highest value was observed in this combination because CMR and NH have high amount of K when compared to RF and CF. Incorporation of *neera* honey in RF, CF, CMR blend significantly (*p* < 0.001) affected the K content.

The value of Na content of the extrudates ranged from 31.21 ± 20.22 mg/100 g to 114.07 ± 4.33 mg/100 g. The highest value was recorded in the combination of F_8_ because NH has rich quantity of Na when compared to other raw materials. The lowest value recorded in the combination of F_1_ because rice contains only 22.83 ± 15.08 mg/100 g of Na. Incorporation of *neera* honey in RF, CF and CMR blend significantly (*p* < 0.001) affected the Na content.

The S content of the extrudates ranged from 37.76 ± 9.58 mg/100 g to 62.72 ± 25.03 mg/100 g in the combination F_4_ and F_1_. This was because rice contains 40.42 ± 9.28 mg/100 g of S, CF contains 58.6 ± 3.39mg/100 g and NH contains 36.75 ± 11.79 mg/100 g of S. Incorporation of *neera* honey in RF, CF and CMR blend significantly affected the S content. In study conducted on coconut haustorium-based extrudates [33], the combination of 100% RF showed the mineral composition as follows: potassium (41.33 ± 2.5 mg/100 g), magnesium (29.22 ± 0.2 mg/100 g), Phosphorus (110.4 ± 2.73 mg/100 g) and calcium (42.02 ± 0.3 mg/100 g).

The Fe content of the extrudates ranged from 2.86 ± 0.85 mg/100 g in F_8_ and 16.20 ± 2.22 mg/100 g in F_3_. The incorporation of NH reduced the Fe content of the extrudates. 

The Mn content of the extrudates ranged from 0.66 ± 0.16 mg/100 g in F_4_ and 1.71 ± 0.17 mg/100 g in F_9_. It was found that incorporation of CMR to the NH extrudate increases the Mn content (Table 12). Incorporation of *neera* honey in RF, CF and CMR blend significantly (*p* < 0.001) affected the Mn content. The Zn content of the extrudates ranged from 0.94 ± 0.56 mg/100 g in F_4_ and 2.15 ± 0.31 mg/100 g in F_1_. It was found out that RF contains Zn that is reflected in extrudates made of it. The lowest value was found in CF extrudates added with water and NH. Incorporation of *neera* honey in RF, CF, and CMR blend significantly affected the Zn content.

The Cu content of the extrudates ranged from 0.540 ± 0.06 mg/100 g in F_1_ to 0.820 ± 0.06 mg/100 g in F_2_ (Table 12). It was found that RF contains high amount of Cu when compared to other raw materials such as CF, CMR and NH. Incorporation of *neera* honey in RF, CF and CMR blend significantly affected the Cu content. Coconut haustorium based extrudates [33] showed that the combination having 100% RF exhibited the following composition: manganese (0.70 ± 0.005 mg/100 g), iron (6.10 ± 0.046 mg/100 g), copper (0.467 ± 0.074 mg/100 g) and zinc (1.22 ± 0.009 mg/100 g).

## 4. Conclusions

The present study was conducted to analyze the nutritional properties of *neera* honey incorporated rice and corn flour extrudates. Infusion of *neera* honey enhanced the nutritional properties, including vitamin C and minerals such as calcium, potassium and sodium, and also improved the antioxidant activity, of the extrudates. It also influenced physical, functional, and color attributes of extrudates resulting in increased bulk density and specific length and reduced expansion ratio and WAI. Likewise, incorporation of coconut milk residue (CMR) has several nutritional benefits, and it contributes to some desirable attributes of the extrudates. The composition of feed is the most important factor that affects the quality of extrudates, and optimizing the moisture level in the composition, flour composition and extrusion processing parameters such as temperature and screw speed of the extruder can result in high quality and nutritious expanded snacks. It can be concluded that incorporation of 60% RF + 25% CF + 15% CMR + 16% FM (NH) based extrudates resulted in nutritious extrudates.

## Figures and Tables

**Table 1:** Composition of rice flour (RF), corn flour (CF), coconut milk residue (CMR) and *neera* honey (NH) combinations and their formulations.

Materials	Formulation
100% RF + 16% feed moisture with water	F_1_
100% RF + 16% feed moisture with NH	F_2_
100% CF + 16% feed moisture with water	F_3_
100% CF +16% feed moisture with NH	F_4_
50% RF + 50% CF + 16% feed moisture with NH	F_5_
50% RF + 50% CF + 16% feed moisture with water	F_6_
60% RF + 25% CF + 15% CMR + 16% feed moisture with NH	F_7_
100% RF + 20% feed moisture with NH	F_8_
85% RF + 15% CMR + 20% feed moisture with NH prepared.	F_9_

**Table 2:** Functional properties and proximate composition of raw materials used for preparation of extrudates.

Parameters	Raw Materials			
	Rice Flour	Corn Flour	Coconut Milk Residue	*Neera* Honey
**Functional properties**				
WAI	2.39^B^ ± 0.05	2.18^B^ ± 0.11	7.12^A^ ± 0.39	Fully soluble
WSI (%)	1.19^D^ ± 0.56	5.87^C^ ± 2.10	12.03^B^ ± 0.12	89.22^A^ ± 0.73
OAI (ml/g)	1.20^B^ ± 0.05	1.21^B^ ± 0.01	1.31^A^ ±0.03	1.30^A^ ±0.05
**Proximate composition**				
Moisture content (% wb)	9.30^B^ ± 0.09	8.38^C^ ± 0.48	5.72^D^ ± 0.04	29.96^A^ ± 0.00
Ash (%)	0.34^B^ ± 0.35	1.37^A^ ± 0.00	0.93^A^ ± 0.06	0.24^B^ ± 0.07
Fat (%)	1.12^B^ ± 1.02	4.24^B^ ± 0.92	44.04^A^ ± 4.96	0.044^B^ ± 0.01
Protein (%)	9.44^A^ ± 1.01	7.291^B^ ± 0.49	7.297^B^ ± 0.21	1.41^C^ ± 0.21

Results are represented as mean ± standard deviation of three replicates. WAI—Water absorption index, WSI—Water solubility index, OAI—Oil absorption index. Values with same letters or no letters are not significantly different at 5% level.

**Table 3:** Biochemical composition of raw materials used for the preparation of extrudates.

Parameters	Raw Materials			
	Rice Flour	Corn Flour	Coconut Milk Residue	*Neera* Honey
**Biochemical analysis**				
Total carbohydrate (%)	92.03^A^ ± 3.11	49.29^B^ ± 2.23	32.09^C^ ± 4.11	30.62^C^ ± 7.26
Total sugar (%)	0.19^B^ ± 0.10	2.73^B^ ± 0.49	1.26^B^ ± 0.01	68.39^A^ ± 6.09
Reducing sugar (%)	0.06^C^ ± 0.00	0.23^B^ ± 0.01	0.10^C^ ± 0.01	1.01^A^ ± 0.04
Total Phenols (mg GAE/100 g)	0.304^B^ ± 0.19	0.386^B^ ± 0.03	0.237^B^ ± 0.07	1.155^A^ ± 0.04
Total Flavonoids (mg QE/100 g)	0.831^C^ ± 0.01	2.400^A^ ± 0.03	1.240^B^ ± 0.23	0.173^D^ ± 0.09
CUPRAC (mg TE/100 g)	1.09^B^ ± 0.25	3.35^A^ ± 0.39	1.22^B^ ± 0.06	1.38^B^ ± 0.48
FRAP (mg TE/100 g)	0.026^C^ ± 0.00	0.318^A^ ± 0.02	0.047^C^ ± 0.00	0.076^B^ ± 0.00
Ascorbic acid (mg/100 g)	2.34^B^ ± 0.00	4.67^B^ ± 0.00	3.51^B^ ± 0.00	39.21^A^ ± 2.48

Results are presented as mean ± standard deviation of three replicates. CUPRAC—Cupric reducing antioxidant capacity, FRAP—Ferric reducing ability of plasma. GAE—Gallic acid equivalent, QE—Quercetin equivalent, TE—Trolox equivalent. Values with same letters are not significant different at 5% level.

**Table 4:** Mineral profile of raw materials used for preparation of extrudates.

Parameters	Raw Materials			
	Rice Flour	Corn Flour	Coconut Milk Residue	*Neera* Honey
**Mineral profile (mg/100 g)**				
**Major minerals**				
Calcium	87.34 ± 0.30	131.34 ± 0.56	196.67 ± 25.69	114.51 ± 42.19
Phosphorus	70.27 ± 5.77	282.57 ± 14.41	97.34 ± 8.45	42.78 ± 1.85
Magnesium	104.81 ± 0.35	26.27 ± 0.11	78.60 ± 30.10	73.62 ± 26.27
Potassium	43.6675 ± 10.34	231.85 ± 3.79	143.75 ± 1.65	528.27 ± 55.26
Sodium	22.83 ± 15.08	130.88 ± 15.33	68.84 ± 30.35	355.21 ± 22.36
Sulphur	40.42 ± 9.28	58.6 ± 3.39	36.25 ± 8.13	36.75 ± 11.79
**Minor minerals**				
Iron	1.48 ± 1.02	5.64 ± 0.18	3.12 ± 1.89	1.30 ± 0.95
Manganese	2.02 ± 1.34	0.85 ± 0.08	3.84 ± 0.16	0.45 ± 0.16
Zinc	2.14 ± 0.33	3.50 ± 0.15	2.20 ± 0.11	3.39 ± 4.21
Copper	0.65 ± 0.02	0.48 ± 0.00	1.10 ± 0.09	0.61 ± 0.13

Results are represented as mean ± standard deviation of three replicates. Values with same letters or no letters are not significant different at 5% level.

**Table 5:** Physical properties of *neera* honey incorporated extrudates.

Extrudate Formulation	Expansion Ratio (ER)	Bulk Density (BD) (g/mL)	Specific Length (SL) (mm/g)
F_1_	3.60^C^ ± 0.13	0.098^F^ ± 0.002	38.20^BC^ ± 0.93
F_2_	1.92^G^ ± 0.01	0.205^D^ ± 0.001	42.21^B^ ± 2.49
F_3_	4.02^A^ ± 0.19	0.079^H^ ± 0.001	33.99^C^ ± 1.37
F_4_	3.38^D^ ± 0.18	0.088^G^ ± 0.00	39.59^BC^ ± 1.01
F_5_	2.86^E^ ± 0.02	0.126^E^ ± 0.002	40.60^BC^ ± 1.12
F_6_	3.78^B^ ± 0.02	0.090^G^ ± 0.002	35.67^BC^ ± 0.42
F_7_	2.33^F^ ± 0.16	0.215^C^ ± 0.004	41.8^BC^ ± 0.82
F_8_	1.91^G^ ± 0.01	0.252^B^ ± 0.002	43.26^B^ ± 3.63
F_9_	1.64^H^ ± 0.09	0.281^A^ ± 0.007	95.51^A^ ± 15.96

Results are presented as mean ± standard deviation of five replicates. Values with same letters are not significantly different at 5% level.

**Table 6:** Functional properties of *neera* honey incorporated extrudates.

Extrudate Formulation	Water Absorption Index (WAI)	Water Solubility Index (WSI) (%)	Oil Absorption Index (OAI) (mL/g)
F_1_	6.17^B^ ± 0.11	13.98^DBC^ ± 1.39	1.55 ± 0.06
F_2_	3.49^CD^ ± 0.12	14.63^BC^ ± 1.23	1.45 ± 0.01
F_3_	7.47^A^ ± 0.25	10.17^DE^ ± 0.85	1.47 ± 0.04
F_4_	5.95^B^ ± 0.01	10.83^DEC^ ± 2.08	1.54 ± 0.03
F_5_	5.65^B^ ± 1.07	11.72^DC^ ± 0.25	1.52 ± 0.03
F_6_	5.80^B^ ± 0.58	7.27^E^ ± 3.55	1.52 ± 0.05
F_7_	3.73^C^ ± 0.10	17.59^B^ ± 1.31	1.41 ± 0.12
F_8_	3.27^CD^ ± 0.23	14.14^DBC^ ± 0.28	1.55 ± 0.17
F_9_	2.61^D^ ± 0.07	24.23^A^ ± 2.28	1.53 ± 0.01

Results are presented as mean ± standard deviation of three replicates. Values with same letters or no letters are not significant different at 5% level.

**Table 7:** Color attributes of *neera* honey incorporated extrudates.

Extrudates Combination	Color Attributes		
	L*	A*	B*
F_1_	57.82^AB^ ± 4.23	−0.57^EF^ ± 0.21	9.99^D^ ± 1.73
F_2_	50.35^C^ ± 2.39	5.11^A^ ± 0.38	15.92^C^ ± 0.72
F_3_	50.52^C^ ± 3.50	−0.09^E^ ± 0.46	21.41^B^ ± 1.57
F_4_	62.46^A^ ± 0.72	2.07^D^ ± 0.45	26.73^A^ ± 0.36
F_5_	57.90^AB^ ± 4.82	2.88^C^ ± 0.55	22.01^B^ ± 2.25
F_6_	56.77^B^ ± 2.59	−1.05^F^ ± 0.47	20.57^B^ ± 2.41
F_7_	54.54^CB^ ± 3.87	3.87^B^ ± 0.75	20.28^B^ ± 1.53
F_8_	42.91^D^ ± 5.36	5.58^A^ ± 0.92	14.92^C^ ± 1.86
F_9_	37.68^E^ ± 2.31	4.90^A^ ± 0.28	11.49^D^ ± 0.80

Results are presented as mean ± standard deviation of five replicates. L—darkness to lightness, A—greenness to redness, B—blueness to yellowness. Values with same letters are not significant different at 5% level.

**Table 8:** Biochemical properties of *neera* honey incorporated extrudates.

Extrudates Combination	Total Carbohydrate (%)	Total Sugar (%)	Reducing Sugar (%)
F_1_	56.49^C^ ± 1.35	4.56^E^ ± 0.92	0.29^C^ ± 0.08
F_2_	65.19^DCB^ ± 2.34	19.82^DBC^ ± 4.55	0.35^C^ ± 0.34
F_3_	80.54^AB^ ± 5.15	1.78^E^ ± 0.55	0.10^C^ ± 0.04
F_4_	78.64^AB^ ± 22.06	17.47^DC^ ± 0.49	0.80^C^ ± 0.38
F_5_	65.32^DCB^ ± 8.15	26.91^B^ ± 4.12	1.11^CB^ ± 0.40
F_6_	63.42^DCB^ ± 7.61	2.30^E^ ± 0.68	0.03^C^ ± 0.01
F_7_	64.10^DCB^ ± 4.11	12.60^D^ ± 0.12	0.21^C^ ± 0.06
F_8_	69.02^CB^ ± 0.15	25.43^BC^ ± 5.96	2.81^A^ ± 1.31
F_9_	63.58^DCB^ ± 0.77	36.82^A^ ± 5.72	2.11^AB^ ± 0.16

Results are presented as mean ± standard deviation of three replicates. Values with same letters are not significant different at 5% level.

**Table 9:** Proximate composition of *neera* honey incorporated extrudates.

Extrudates Combination	Moisture Content (%w.b)	Ash (%)	Fat (%)	Protein (%)
F_1_	6.38 ± 1.15	0.29 ± 0.13	1.42 ± 0.81	7.99 ± 0.14
F_2_	5.56 ± 0.07	0.68 ± 0.41	1.14 ± 0.35	7.97 ± 0.01
F_3_	8.56 ± 1.37	0.54 ± 0.07	2.14 ± 1.13	8.77 ± 0.45
F_4_	6.47 ± 0.91	0.59 ± 0.00	1.52 ± 0.81	8.33 ± 0.24
F_5_	6.44 ± 1.37	0.74 ± 0.35	1.74 ± 0.99	8.20 ± 0.09
F_6_	10.61 ± 3.17	0.39 ± 0.00	1.94 ± 1.34	8.24 ± 0.02
F_7_	3.76 ± 0.01	0.34 ± 0.06	4.90 ± 0.75	9.23 ± 1.64
F_8_	6.63 ± 0.88	0.89 ± 0.13	4.96 ± 2.72	7.69 ± 0.12
F_9_	6.25 ± 2.12	0.68 ± 0.13	3.20 ± 0.61	7.51 ± 0.14

Results are presented as mean ± standard deviation of three replicates. Values with no letters are not significant different at 5% level.

**Table 10:** Phenolics, flavonoids, vitamin C and antioxidant properties of *neera* honey based extrudates.

Extrudates Combination	Phenolic Content (mg GAE/100 g)	Flavonoid Content (mg QE/100 g)	Vitamin C (mg/100 g)	Antioxidant Potential	
				CUPRAC (mg TE/100 g)	FRAP (mg TE/100 g)
F_1_	0.178^DB^ ± 0.01	0.361^B^ ± 0.11	4.70^DE^ ± 0.01	1.11^EF^ ± 0.47	0.048^D^ ± 0.007
F_2_	0.197^CDB^ ± 0.01	0.177 ^D^ ± 0.02	8.78^C^ ± 0.83	0.80^F^ ± 0.13	0.068^CAB^ ± 0.005
F_3_	0.161^D^ ± 0.03	0.528^A^ ± 0.00	2.34^F^ ± 0.01	2.97^A^ ± 0.27	0.072^AB^ ± 0.006
F_4_	0.171^CD^ ± 0.04	0.303^BC^ ± 0.00	5.85^D^ ± 0.01	2.30^B^ ± 0.04	0.076^A^ ± 0.002
F_5_	0.212^CDB^ ± 0.04	0.211^DC^ ± 0.04	7.61^C^ ± 0.80	2.09^BC^ ± 0.29	0.067^CB^ ± 0.001
F_6_	0.188^CDB^ ± 0.02	0.245^DC^ ± 0.03	3.51^FE^ ± 0.00	1.89^DBC^ ± 0.12	0.061^C^ ± 0.001
F_7_	0.359^A^ ± 0.10	0.180^D^ ± 0.04	14.02^B^ ± 0.03	1.24^DEF^ ± 0.49	0.069^CAB^ ± 0.001
F_8_	0.277^CAB^ ± 0.05	0.208^DC^ ± 0.00	17.58^A^ ± 1.62	1.49^DEC^ ± 0.03	0.070^AB^ ± 0.00
F_9_	0.286^AB^ ± 0.03	0.201^DC^ ± 0.03	17.59^A^ ± 0.07	1.26^DEF^ ± 0.16	0.076^A^ ± 0.00

Results are presented as mean ± standard deviation of three replicates GAE—Gallic acid equivalent, QE—Quercetin equivalent, TE—Trolox equivalent; CUPRAC—Cupric reducing antioxidant capacity, FRAP—Ferric reducing ability of plasma. Values with same letters are not significantly different at 5% level.

**Table 11:** Major mineral composition of *neera* honey incorporated extrudates.

Extrudates Combination	Ca (mg/100 g)	P (mg/100 g)	Mg (mg/100 g)	K (mg/100 g)	Na (mg/100 g)	S (mg/100 g)
F_1_	97.74^B^ ± 22.15	75.94^BC^ ± 20.05	97.65^A^ ± 12.76	46.84^E^ ± 3.83	31.21^B^ ± 20.22	62.72 ± 25.03
F_2_	151.58^A^ ± 23.85	69.47^C^ ± 11.34	84.40^AB^ ± 24.34	102.86^CDB^ ± 15.78	49.67^B^ ± 27.82	45.77 ± 18.69
F_3_	184.80^A^ ± 22.02	86.90^B^ ± 6.23	65.10^AB^ ± 44.96	100.01^CD^ ± 16.60	48.70^B^ ± 15.10	42.24 ± 29.47
F_4_	152.42^A^ ± 24.07	87.38^B^ ± 7.17	52.30^B^ ± 0.53	117.60^CAB^ ± 4.10	60.24^B^ ± 15.71	37.76 ± 9.58
F_5_	108.61^B^ ± 25.79	79.86^BC^ ± 8.17	91.05^A^ ± 14.42	100.86^CDB^ ± 4.96	48.19^B^ ± 24.42	46.80 ± 17.42
F_6_	152.07^A^ ± 24.19	80.08^BC^ ± 10.63	65.30^AB^ ± 15.52	91.29^D^ ± 19.06	42.29^B^ ± 12.67	56.30 ± 17.12
F_7_	173.33^A^ ± 2.43	79.42^BC^ ± 5.38	52.00^B^ ± 0.73	110.59^CAB^ ± 6.03	53.63^B^ ± 20.34	48 ± 20.70
F_8_	173.83^A^ ± 0.85	103.16^A^ ± 2.24	91.23^A^ ± 14.80	119.02^AB^ ± 9.78	114.07^A^ ± 4.33	52.78 ± 16.86
F_9_	152.06^A^ ± 24.84	87.58^B^ ± 4.08	91.24^A^ ± 14.90	126.93^A^ ± 10.56	107.64^A^ ± 11.32	44.61 ± 8.65

Results are presented as mean ± standard deviation of three replicates.Ca—Calcium, P—Phosphorus, Mg—Magnesium, K—Potassium, Na—Sodium, S—Sulphur.Values with same letters are not significantly different at 5% level.

**Table 12:** Trace mineral composition of *neera* honey incorporated extrudates.

Extrudates Combination	Fe (mg/100 g)	Mn (mg/100 g)	Zn (mg/100 g)	Cu (mg/100 g)
F_1_	9.42^BC^ ±1.71	1.31^BC^ ± 0.16	2.15^A^ ± 0.31	0.540^B^ ± 0.06
F_2_	5.65^D^ ± 3.77	1.17^DC^ ± 0.04	1.85^AB^ ± 0.34	0.820^A^ ± 0.06
F_3_	16.20^A^ ± 0.22	0.73^E^ ± 0.14	1.29^CB^ ± 0.12	0.545^B^ ± 0.10
F_4_	16.05^A^ ± 2.22	0.66^E^ ± 0.16	0.94^C^ ± 0.56	0.552^B^ ± 0.22
F_5_	11.09^B^ ± 0.33	0.96 ^D^ ± 0.18	1.36^CB^ ± 0.51	0.560^B^ ± 0.15
F_6_	14.31^A^ ± 1.40	1.17 ^DC^ ± 0.14	1.47^CB^ ± 0.21	0.757^AB^ ± 0.19
F_7_	7.21^DC^ ± 1.09	1.47^B^ ± 0.22	1.38^CB^ ± 0.40	0.560^B^ ± 0.14
F_8_	2.86^E^ ± 0.85	1.26^BC^ ± 0.13	1.43^CB^ ± 0.33	0.542^B^ ± 0.11
F_9_	7.68^DC^ ± 1.10	1.71^A^ ± 0.17	1.53^CB^ ± 0.26	0.677^AB^ ± 0.15

Results are presented as mean ± standard deviation of three replicates. Fe—Iron, Mn—Manganese, Zn—Zinc, Cu—copper. Values with same letters are not significantly different at 5% level.

## Data Availability

Data are available from the author R.P.
